# Operationalizing the ECOWAS regional one health coordination mechanism (2016–2019): Scoping review on progress, challenges and way forward

**DOI:** 10.1016/j.onehlt.2021.100291

**Published:** 2021-07-08

**Authors:** Virgil Kuassi Lokossou, Nnomzie Charles Atama, Serge Nzietchueng, Bernard Yao Koffi, Vivian Iwar, Nadia Oussayef, Chukwuma David Umeokonkwo, Casey Barton Behravesh, Issiaka Sombie, Stanley Okolo, Edgard-Marius Ouendo

**Affiliations:** a ECOWAS Regional Centre for Disease Surveillance and Control, Nigeria; b Department of Livestock Services, Kaduna State Ministry of Agriculture & Forestry, Nigeria; c Department of Viroscience, Erasmus Medical Center Rotterdam, Netherlands; d Food and Agriculture Organisation of United Nations, Canada; e Faculty of Veterinary Medicine, University of Liège, Belgium; f ECOWAS Directorate of Environment, Nigeria; g ECOWAS Regional Animal Health Center, Nigeria; h US Centres for Disease Control and Prevention, USA; i Department of Community Medicine, Alex Ekwueme Federal University Teaching Hospital, Abakaliki, Ebonyi State, Nigeria; j Institut Régional de Santé Publique, Benin; k West African Health Organization, Nigeria

**Keywords:** One health, ECOWAS, Regional one health coordination mechanism, West Africa, Multisectoral coordination, OH, One Health, ECOWAS, Economic Community of West African States, WAHO, West African Health Organization, RAHC, ECOWAS Regional Animal Health Center, WHO, World Health Organization, OIE, World Organization for Animal Health, USAID, United States Agency for International Development, FAO, United Nation Food and Agricultural Organization, R-OHCM, Regional One Health Coordination Mechanism, OHZDP, One Health Zoonotic Diseases Prioritization, RCSDC, ECOWAS Regional Centre for Surveillance and Disease Control, ECO-WARN, ECOWAS Early Warning Response Network, CDC, US Centers for Disease Control and Prevention, NPHI, National Public Health Institutes

## Abstract

Based on recommendations from two consultative meetings held in Dakar, Senegal (2016) and Abuja, Nigeria (2017) the Economic Community of West African States (ECOWAS) implemented a Regional One Health Coordination Mechanism (R-OHCM). This study analyzed the process, challenges and gaps in operationalizing the R-OHCM in West Africa. We utilized a scoping review to assess five dimensions of the operation of an R-OHCM based on political commitment, institutional structure, management and coordination capacity, joint planning and implementation, as well as technical and financial resources. Information was gathered through a desk review, interview of key informants, and the viewpoints of relevant stakeholders from ECOWAS region during a regional One Health technical meeting in Lomé, Togo in October 2019. It was found that political commitment at regional meetings and the countries adoption of regional frameworks were key strengths of the R-OHCM, although there are continued challenges with commitment, sustainability, and variability of awareness about One Health approach. ECOWAS formulated regional strategic documents and operationalized the One Health secretariat for strengthening coordination. The R-OHCM has technical working groups however, there is need for engagement of more specialized workforce and a harmonized reporting structure. Furthermore, inadequate focus on operational research, and weak national OHCM are identified as main gaps. Finally, the support of technical and financial partners will help to address the lack of funding which limits the implementation of the R-OHCM. West Africa has demonstrated profound effort in adopting the One Health approach at regional level but is presently deterred by challenges such as limited skilled One Health workforce, especially in the animal and environmental health sectors, and access to quality of One Health surveillance.

## Highlights

• ECOWAS region has demonstrated political will to adopt and operationalize one health approach for global health security
• The region needs specialized and competent one health workforce, funding, and committed policymakers at national levels
• Supportive leadership and advocacy will engage all sectors to collaborate in operating one health agenda.
• Strengthened One Health capacities in institutions will enhance the operationalization of the regional One Health approach
• ECOWAS RCSDC and National Public Health Institutes should serve as One Health secretariat at regional and national level

## 1 Introduction

Recent outbreaks of zoonotic diseases and the growing concern on antimicrobial resistance (AMR) in West Africa have increasingly revealed the importance of multisectoral and multidisciplinary collaboration and coordination as promoted by International Health Regulations (IHR 2005). One Health (OH) is defined as a collaborative, multisectoral, and transdisciplinary approach working at the local, subnational, national, regional and global levels with the goal of achieving optimal health outcomes through recognizing the interconnection between people, animals, plants, and their shared environment [1]. The OH approach is now well-recognized in global health security efforts in terms of its potential to contribute to better preparedness for prevention, detection and mitigation of the impacts of infectious diseases (both emerging, re-emerging and endemic) as well as non-communicable diseases [2,3].

In the attempt to foster multisectoral collaboration and coordination by operationalizing one health practices in the Economic Community of West African States (ECOWAS) region, a high level regional consultative meeting was jointly organized in Dakar, Senegal in 2016 by a consortium of partners from multiple sectors including West African Health Organization (WAHO), ECOWAS Regional Animal Health Center (RAHC), ECOWAS Directorate of Education, Culture, Science and Technology, World Health Organization (WHO), World Organization for Animal Health (OIE), United States Agency for International Development (USAID) - Regional Office of West Africa, World Bank Group and United Nation Food and Agricultural Organization (FAO) [4,5]. The meeting aimed at sensitizing participants on importance and need for a OH approach and at developing regional agenda for institutionalizing One Health approach in West Africa. The recommendations of the Dakar Consultation required ECOWAS Member States to design a robust regional and national One Health Coordination Mechanisms (OHCM) and sustain partnerships to achieve optimal health security in West Africa [4].

In 2017, WAHO convened a high level political and technical meeting in Abuja, Nigeria with the representatives from Ministries of Health, Agriculture and Environment from the 15 Member States as well as major technical and financial partners [6]. The meeting was to implement one of the recommendations of the Dakar consultative meeting on establishing a regional framework for the R-OHCM. The Ministers reaffirmed the critical need for ECOWAS Member States and the region to engage in one health approach implementation and build capacities for better prevention, detection, and response to emerging and re-emerging disease threats. Furthermore, Abuja meeting also identified the need to develop strategies to support national one health initiatives and a regional level coordination mechanism. The call for technical and financial partners to support and work through the existing structures for institutionalizing the one health approach in the region was encouraged [6]. In 2018, the region conducted a sub-regional One Health Zoonotic Disease Prioritization (OHZDP) workshop in Dakar, Senegal with the technical support of the US CDC and FAO and cumulated with a prioritization of seven important endemic and emerging zoonotic diseases including anthrax, rabies, Ebola and other haemorrhagic fevers, zoonotic influenza, trypanosomiasis, and yellow fever [7].

While some progress has been made at national levels through the support of regional bodies, challenges still exist in ensuring an effective collaboration and coordination at the regional level across the key sectors. The purpose of this paper is to document and summarize the key achievements and identify the gaps in policy and practice to support the development of R-OHCM in West Africa. These findings will provide insights to leaders and policy makers on where to invest time and resources to build the effective multisectoral coordination mechanisms in other regions around the world.

## 2 Methods

We conducted a scoping review to examine the operationalization of the ECOWAS R-OHCM[8]. In addition, a consultation meeting was conducted and more information were elicited through interviews with key informants and a panel discussion.

### 2.1 Phase 1: Desk review

We conducted a literature search of published (peer reviewed journal articles) and grey literature (technical reports, meeting and conference reports, position papers) to explore progress, challenges, opportunities and threats over 2016–2019 period. We searched in electronic databases including Google Scholars, PubMed, African Journals online, Web Science, ECOWAS web pages for OH articles and published activities on OHCM in West Africa at regional perspective from the years 2016 to 2019. The search yielded more unpublished documents (*n* = 20) as summarized in Table 1 with the ones most relevant to the ECOWAS R-OHCM.

**Table 1:** Summary of most relevant documents related to ECOWAS R-OHCM.

Title	Year(s)
One Heath technical in Dakar technical report	2016
One Heath Ministerial Meeting in Abuja technical report	2017
Global Security Agenda Annual Report	2016, 2017, 2018, 2019
Evaluation of One Health platform in West Africa	2018
Annual reports of the West African Health Organization	2016, 2017, 2018 2019
Strategic Action Plans for combating HPAI virus	2019
reports from the directorate of environment Situational analysis report of	2019
ECOWAS regional strategic preparedness and response plan	2018
National One Health Bridging workshops of Niger	2019
National One Health Bridging workshops of Benin	2019

Two authors (VL and NA) reviewed all the documents from the search to determine which are relevant to address the objectives of the study. We summarized the strengths, gaps, opportunities and threats for the operationalization of one health regional coordination mechanism, which was then further validated by a third author (SNSN).

### 2.2 Phase 2: Consultation phase

Building on the desk review, we conducted a series of interviews with key informants within ECOWAS Commission, and a panel discussion with national and international stakeholders including one health contributors operating at regional level in West Africa during a one health technical meeting held at Lomé, Togo in 2019 to gather stakeholders' opinions on one health implementation and future directions.

#### 2.2.1 Interview of key informants

In order to describe the R-OHCM, six key informant interviews were conducted. The participants in the interview were purposively selected based on their current job descriptions in ECOWAS Commission and their active roles and routine involvement in one health operationalization in the region. Two of the representatives were from the West African Health Organization and one each from ECOWAS Regional Centre for Surveillance and Disease Control (RCSDC), RAHC, ECOWAS directorate of high education, and ECOWAS Directorate of Environment. The interviews were conducted using an interview guide. Each interview lasted around one hour. The interviews were recorded and transcribed within 24 h of interview. Thematic manual analysis was conducted.

#### 2.2.2 Panel discussions

The WAHO in collaboration with the RCSDC and the ECOWAS Regional Animal Health Center (RAHC) jointly organized a one health technical meeting at Lomé in late October 2019 to discuss the status of the implementation of one health approach at regional level in West Africa.

During this meeting, two participatory panel discussions were conducted with representatives (from the three main sectors including human, animal, and environment) of 15 ECOWAS countries, as well as 28 stakeholders from academia, African tripartite secretariat composed of WHO, OIE, and FAO and other relevant technical and financial partners. A participatory panel discussion was referred to as an organized public exchange of ideas with experts and other participants discussing a particular topic to create an action map and incorporate directions for possible future actions [[23]]. Contributions to the panel discussions came from 140 participants who were selected based on the following criteria:

• Moderator: resource persons selected based on their experience in collaboration with the R-OHCM
• Panellist: Experts from various technical and financial organizations supporting the R-OHCM including WHO, OIE, FAO, USAID, Africa CDC, German Technical Cooperation (GIZ) World Bank, and EcoHealth Alliance
• Audience: National OHCM contributors, Non-governmental Organizations, experts, academia and others

Each thematic point discussed was raised by the moderator who facilitated the discussions. The points were first discussed among panellists before opening to the larger audience for comments and contributions.

The panel discussion provided additional source of information and perspectives to the preliminary findings. The information provided by the subject matter experts during the panel discussion also revealed the level of functionality of the one health approach, while providing likely future interventions on the regional one health agenda. The panel members discussed in-depth how to effectively support ECOWAS in the implementation of the R-OHCM, and on the best practices to strengthen multisectoral collaborations at regional and national levels. The entire discussions lasted about six hours. From all participants, consents were obtained but anonymity was not guaranteed. However, confidentiality and privacy were maintained. The decision on points considered as either progress, gaps, challenges, opportunities and threats was arrived at through participatory approach by the moderators of the panel discussions.

## 3 Findings

The findings of this scoping review were summarized through an analytic framework based on five dimensions adapted from the criteria used to assess institutionalization and operationalization of one health at the national levels [,^15^]. Although the dimensions are often used to assess one health coordination mechanism (OHCM) at national levels [], they were re-defined (Table 2) to assess the regional one health coordination mechanism (R-OHCM) []. (See Table 3.)

**Table 2:** Definition and Components of the ECOWAS OH Coordination Mechanism [15].

Dimensions	Definition	Components
Political commitment	Political commitment: the actions, events, and factors that motivate stakeholders to take concerted action toward establishing and sustaining the regional One Health coordination mechanism	• Promotion of shared goals among sectors • Existence of Regional OH Advocacy Strategy • Availability of OH contributors • Existence of OH risk communication group based on OH approach, • Existing national or regional supportive bodies • Enabling Environment: Global Health Security Agenda, International Health Regulations (IHR 2005), Adoption of Regional Framework, Performance of Veterinary Services (PVS) and IHR-PVS National Bridging Workshop
Institutional structure	How the regional level is organizing its One Health coordination mechanisms, including the legal mandate, framework, duties and obligations, lines of authority, and reporting framework.	• Existence of regional and formal OH Structure • Establishment and engagement of relevant organizations for supporting the operationalization of the R-OHCM • Development of laws and regulations
Management and coordination capacity	The ability to convene partners, meet management and technical standards, monitor and measure progress toward health security objectives, and sustain the commitment.	• Existence of strategic framework and its adoption of the OH approach in ECOWAS region • Leadership and coordination capacity o Existence of Technical Working Groups (TWGs) o Knowledge management framework (Knowledge transfer and use of evidence to improve implementation of OH) • Engagement of stakeholders in the implementation of OH interventions Communication and information sharing including communication protocols
Joint planning and implementation	The engagement of stakeholders to develop national roadmaps, design plans of action, conduct simulations, and manage emerging, re-emerging and endemic infectious diseases investigations.	• Joint Planning o Regional OH strategic planning o Regional OH Zoonotic Disease Prioritization o Regional AMU &Antimicrobial resistance o Regional food security and food safety o Regional preparedness and response planning o Regional risks assessment and management • Joint Implementation o Roadmaps o Routine vigilance and information sharing o Routine surveillance and information sharing o Preparedness for, Prevention to, detection, and response to infectious diseases outbreaks o Regional level information sharing/meetings o Regional simulation exercises
Technical and financial resources	The identification and mobilization technical, and financial resources needed to operate and sustain the regional coordination mechanisms 24	• Technical Resources: o Availability of adequate and skilled workforce o Availability of tools • Financial domestic and partners Resources: ECOWAS domestic fund, REDISSE (a grant at sub-regional and loan at country level), Regional Pandemic Preparedness Project /GIZ, Fleming fund,

**Table 3:** Progress in implementation of the ECOWAS regional OH Coordination Mechanism across key technical areas (2016 to 2019).

Technical component of Framework	Key achievements/Progress
Political commitment and Leadership	• Commitment of technical and financial partners to support National OH Coordination mechanisms in ECOWAS member states • OH Secretariat within the ECOWAS commission established and operationalized • 2 regional policy debates held with ministers from all relevant sectors during Political meetings (2016 and 2017) for strategic deliberations and orientations of the R-OHCM • Strategic regional documents for supporting the implementation of OH regional coordination mechanisms adopted o ECOWAS regional strategic preparedness and response plan highlighting key strategies for implementing OH approach o Supplementary Act A/SA.4/12/08 adopting an ECOWAS Environmental Policy; o Regional vulnerability reduction and climate change adaptation programme o Regional forest Convergence Plan o Regional Strategies and an integrated plan on Chemical & Hazardous Wastes management; o Regional Biosafety regulation; o A regional strategy to Combat Illegal Trade in Endangered Wildlife Species. o Regional Strategy for Control & Elimination of Dog Transmitted Rabies o Strategic Action Plan for controlling Highly Pathogenic Avian Influenza
Institutional Structure within ECOWAS	• Central coordination under the leadership of the President of ECOWAS Commission • ECOWAS Regional Centre for Disease Surveillance and Control (RCDSC) established and serves as the OH Secretariat, Regional Laboratory Networks (RLN) established, ECOWAS Regional Rapid Response Team (RRT), West African Network of Infectious Disease Surveillance, Risk communication specialist networks and other relevant networks in RCDSC also functional • Regional Animal Health Centre (RAHC) established and functional to coordinate animal health activities • ECOWAS Department of Environment (EDE) established to coordinate environmental sector activities • Early Warning Department (EWD) established for all hazards • ECOWAS social and Humanitarian affairs established for supporting the coordination of the humanitarian response • Establishment of other departments in charge of trade, finance, and other resources • Adoption of laws and regulations o Health safety of Plants, animals & food (Reg. C/REG.21/11/10) o Law for the Management of veterinary committee (Reg C/REG.22/11/10); o Supplementary Act A/SA.4/12/08 adopting an ECOWAS Environmental Policy;
Management and Coordination Capacity	• Three routine outcome-oriented meetings for strengthening coordination mechanisms at regional level were conducted with the participation of all • ECOWAS RCSDC governance manual and strategic plans approved, and staff recruited into different units. • Regional Strategy for the Control and diagnosis and mapping of Rift Valley Fever (RVF) spread in Guinea, Liberia and Sierra Leone developed • Pilot Actions for the Integrated Control of Trypanosomiasis with trapping of Tse-tse flies and treatment elaborated • Monitoring progress of the regional OH agenda toward Health security objectives during technical meetings conducted • Technical and financial support provided by ECOWAS for the development and implementation of strategic document (policies, plans) • Provide technical and financial support to the 15 Member States and strengthen national OH system management capacities • Assessment of migration to District Health Information Software -DHIS2 and Interoperability system implementation has been done • OH Bulletins: Epidemiological Bulletin printed monthly
Joint planning and implementation	• Regional OH Strategic plan development in process • IHR-PVS National Bridging workshops completed (in Senegal, Guinea, Liberia and Sierra Leone) in collaboration with OIE-WHO • Risk assessment and vulnerability mapping in ECOWAS region: Joint Risk Assessments conducted through the vulnerability risk • ECOWAS Regional RRT trained in September 2016 and ready to deploy as needed • Joint response and Simulation exercise conducted • Technical working group on AMR established (the three relevant sectors involved) • Technical working group on research and training and knowledge management (Reinforcement of Regional Epidemiological Surveillance (RESEPI) and Laboratory Diagnosis (RESOLAB) Networks on Knowledge Management) • OH champions in ECOWAS region identified and trained • Field Epidemiologists trained through the FETP programs • Technical meeting held on the roles of National Public Health Institutes (NPHIs) • Regional TWG in thematic areas: Zoonotic diseases, AMR, vector-borne diseases, food and water safety issues, data sharing (Health information) established and operational • Protocol for monitoring entomological indicators of arboviruses including zoonosis developed • Regional OH Zoonotic Disease Prioritization conducted • Capacity of reference laboratories strengthened (with 2 animal health labs) • Implementation of National Public Health Institutes /National Coordinating Institutions across Member States supported • Organization of OH Day: celebration of OH initiatives in ECOWAS region • Health information Systems capacity strengthened: through training, distance assistance for integration of animal health and human health databases • Dissemination of information: Production of Joint Bulletins
Technical and financial resources	• ECOWAS partners forum meeting to mobilize financial and technical resources annually • OH in-service and pre-service technical capacity building conducted • Epidemic funds to combat epidemic prone diseases including zoonoses established

### 3.1 The ECOWAS regional one health coordination mechanism

The ECOWAS regional one health Coordination mechanism (R-OHCM) framework was adopted in July 2017. Its leadership is under the President of the ECOWAS Commission, and the relevant statutory appointees whose departments and institutions are involved in the framework of the operationalization of the R-OHCM [6]. The RAHC collaborates with the RCSDC as a coordination hub for trans-boundary animal disease (TADs) and emerging threats from zoonosis to foster an effective OH collaboration in the region. Working in collaboration with RCSDC and RAHC, the ECOWAS Directorate of Environment and natural resources, the ECOWAS Directorate of Education, Culture, Science and Technology, also play key roles in R-OHCM for prevention, surveillance, early warning, detection, and response to public health threats [[10], [11]].

**Fig. 1:**
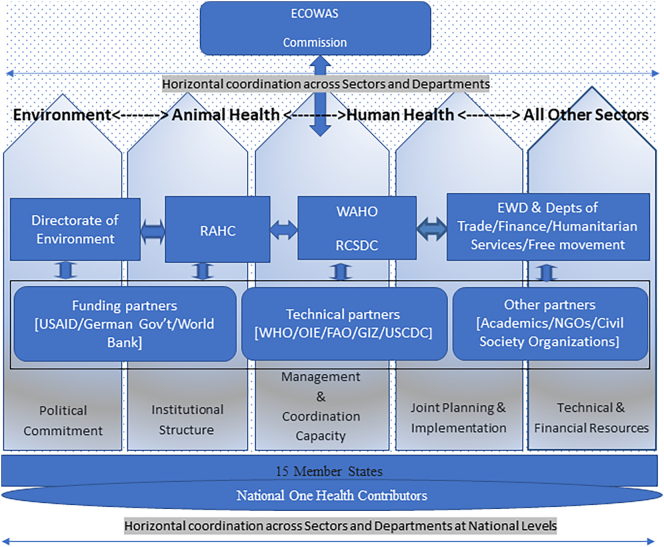
ECOWAS Regional OH Platform and Coordination Structure. **Footnote. (*EWD; Early Warning Department, RAHC; regional animal health centre, WAHO; West African Health Organization, RCDSC; Regional Center for Disease Surveillance and Control, USCDC; United States Centres for Disease Control and Prevention, GIZ; Deutsche Gesellschaft fur Internationale Zusammenarbeit, NGOs; Non-governmental Organizations. Note: National OH Contributors refers to all public health institutes and actors at the national level including National and regional reference laboratories resident in the member states.*) NOTE: Colour should be used for figure in print.

R-OHCM also leverages on the crisis preparedness activities of the ECOWAS Early Warning Response Network (ECO-WARN); an early warning system established under the Early Warning Department (EWD) to prevent, manage and resolve conflicts and large scale emergencies and to promote peace and security within the region [11]. Other critical supporting departments at the ECOWAS Commission include Directorate of Education, Culture, Science and Technology, Directorate of Finance, Directorate of early warning, Directorate of Trade, as well as the Directorate of Humanitarian Affairs that are also involved in the R-OHCM to support advocacy and resource management during crisis. All these actors with the support of financial and technical partners are working together to ensure efficient use of existing resources (Fig. 1). From 2016 to 2019, the R-OHCM made considerable progress through several interventions as listed in the Table 3 based on the different key dimensions.

### 3.2 Identified strengths, challenges, opportunities, and threats of the ECOWAS R-OHCM

The results of this study are presented in the form of strengths, gaps, threats, and opportunities of the R-OHCM according to the five dimensions for institutionalizing an OHCM (Table 4).

**Table 4:** Scoping analysis outcome on operationalization of ECOWAS Regional OH Coordination Mechanism across all relevant sectors.

	*Strengths*	*Challenges*	*Opportunities*	*Threats*
Political commitment and Leadership	• Political commitment at Dakar meeting • Keen interest of Member States in adopting the OHCM plans • Support to existing regional institutional structures	• Unsustainable commitment by policy makers • Lack of overall awareness about OH Within ECOWAS High turn-over of political appointees in the ECOWAS region	o Willingness of technical partners to support OH o Potential to create budgetary planning for OH	o Political instabilities in some countries thus affecting government commitments into the regional OHCM initiatives
Institutional Structure within ECOWAS	• Institutionalization: adoption of regional OH Framework by the ECOWAS Council of Health Ministers • Appointment of WAHO-RCDSC as the Secretariat • Established regional institutions to support the coordination of regional OH platform (WAHO, RCDSC, RAHC, ECOWAS Early warning department, ECOWAS Social and Humanitarian Affairs, ECOWAS Directorate of Environment) • A developed regional framework for OH approach	• Lack of protocols for sharing information, including what information to share and when, to neighbouring countries both before and during a public health emergency • Lack of policy Guidelines and governance manuals for the regional OH Coordination mechanism • No harmonized surveillance and reporting document for the region based on OH approach	• Existence of regional prioritized list of zoonotic diseases for strengthening collaborations among institutions	• Lack of adoption of the list of prioritized diseases for implementation
Management and Coordination Capacity	• Existence of regional networks and technical work groups (Taskforces) to support the operationalization of regional OH platform • Existence of ECOWAS Inter Institutional strategy based on OH approach • Existence of risk communication strategy based on OH Approach • Existence of priority zoonotic diseases at the regional level and an OHZDP report describing plans to address these priority zoonotic diseases for ECOWAS region • Ad-hoc collaboration between institutions and actors at regional level	• Lack of skilled workforce in all sectors to support the operationalization of OH Approach • Lack of strategic plan for Regional OH Platform with clear priorities for OHCM in ECOWAS region • Lack of Monitoring & Evaluation mechanism for the implementation process • Lack of Monitoring & Evaluation mechanism for the implementation process • Lack of Taskforces for ECOWAS regional Priority Zoonotic Diseases • Inadequate OH capacity and orientation in the animal and environmental health • Lack of advocacy tools: inefficient and insufficient advocacy and message development from OH actors • Poor focus on operational research • Lack of a well-established recognition and rewarding structure for OH initiatives at regional level • Weak multisectoral collaborations lacking the engagement of all relevant sectors in key activities	• Countries with established OH Platforms can be benchmarked by others • Operation of the RAHC with the potential to strengthen capacity on animal health	• High turn-over of technical experts at the country level
Joint planning and implementation of OH strategies	• IHR-PVS workshop for joint planning and reporting • Existence of priority zoonotic diseases at the regional level and an OHZDP report describing plans to address these priority zoonotic diseases for ECOWAS region • Establishment of the regional reference laboratory network involving the participation of Animal health laboratories • Establishment of Joint Regional Rapid Response Teams for addressing zoonotic diseases with Joint training and Simulation exercises • Strengthening interoperability between Human Health and animal health system through a DHIS-2 (Joint trainings, SOP SIMEX and other collaborative initiatives)	• Data collected by individual sectors –however lack of integrated risk analysis/sharing with involvement of key partners at regional level • Weak surveillance and response system • Lack of joint implementation plans	• Networks of Universities and training schools to support OH capacity building and operational research • Existing OH technical bodies for supporting the operational of R-OHCM • Establishment of specialized technical working groups and taskforces	o Lack of involvement of animal and environment health sectors at country level
Technical and Financial Resources	• Existing projects in ECOWAS region such as REDISSE (World Bank) and CAPS (USAID) which is currently supporting OH • ECOWAS domestic funding committed for supporting the operationalization of the R-OHCM	• Lack of funding solely dedicated to the ECOWAS regional OHCM	o Existence of funding mechanisms in ECOWAS region such as: o REDISSE funds to support capacity building activities, surveillance and laboratory capacity o Leadership and capacities strengthening project o GHSA DFID/TDDAP o Fleming funds o DTRA o Regional Pandemic Preparedness Project /GIZ o Priorities of funders shift to single diseases during emergency situations	o Lack of sustainability of funding sources and lack of complete ownership in-countries

The observed commitment of countries to adopt an OH approach in *Political commitment and leadership* framework during the Dakar meeting and their keen interest in adopting the OHCM framework for the region was an identified strength of the R-OHCM. Unsustainable commitment by the national policy makers coupled with the poor overall awareness of technical and political stakeholders on the OH collaboration within the region was identified as a gap. Furthermore, the periodic turn-over among ECOWAS leaders, as well as a lack of budgetary planning for OH presents a challenge that hampers the rate and speed of implementation of OH activities. Political disturbances in some of the Member States is a factor likely to delay the R-OHCM implementation. However, the R-OHCM can leverage on the availability and willingness of regional and international partners to support its full implementation.

The R-OHCM was found to already have an *institutional structure* for multisectoral coordination with existence of a regional OH Framework and an established One Health Secretariat (around ECOWAS commission infrastructure) comprising of representatives from the major components including WAHO, RCDSC, RAHC and the Directorate of Environment. The challenges included the lack of harmonized protocols and framework for collecting and sharing information among Member States before, during and after a public health incidence or emergency, and the lack of policy guidelines and governance manual for the R-OHCM.

The existence of a One Health based inter-institutional communication and risk communication strategy, and a collaboratively developed list of priority zoonotic diseases and associated action plans through the ECOWAS OHZDP workshop, alongside strong regional networks and taskforces that support the operationalization of the ECOWAS R-OHCM were considered strengths of the platform in terms of its *Management and Coordination Capacity*. Existing taskforces include: (i) surveillance and health information systems; (ii) preparedness and response including coordination; iii) training and research; (iv) laboratory and antimicrobial resistance; (v) animal health priorities; and (vi) environmental health issues. Ad-hoc collaboration between institutions and actors at the regional level during public health emergencies was also identified as a strength of the R-OHCM with several areas for improvement also summarized (Table 4). The R-OHCM is also leveraging on established platforms such as the availability of the National Public Health Institutions (NPHIs) for further growth [12].

In an effort to achieve true multisectoral collaboration through *joint planning and implementation* of OH strategies by the region, International Health Regulation – Performance Veterinary Services (IHR-PVS) National Bridging Workshops have been conducted in seven ECOWAS counties including Nigeria, Niger, Benin, Liberia, Sierra Leone, Senegal and Guinea in collaboration with WHO and OIE to ensure a unified reporting system for diseases [13]. Areas to improve upon for the future included the development of surveillance and reporting system with integrated risk analysis and data sharing among key partners at the regional level and more involvement of the animal health and environment sectors. The network of universities and training institutions across the region was identified as an opportunity to support one health capacity building and research initiatives. However, the lack of involvement of the animal health and environment sectors at country level in implementing OH activities is a threat to achieving the appropriate R-OHCM.

Regarding the *technical and financial resources*, strengthening domestic funding mechanisms was identified as the key priority for sustaining the OH collaboration and coordination within the region, however other funding from existing projects are available and utilized. The Regional Disease Surveillance Enhancement (REDISSE) funds from the World Bank is opened to support integrated surveillance and Health information systems, laboratory capacities and networks, preparedness and response capacity and building for adequate workforce both at regional and national level. The region has also benefited from the other specific funds from United States Agency for International Development (USAID) project, United Kingdom's Department for International Development (DFID), Regional Pandemic Preparedness Program (RPPP/GIZ) by the German development agency (GIZ) and European Union, and the Fleming funds [25]. Furthermore, the shift in priorities of funding partners to a single disease during emergencies is also leveraged upon by the R-OHCM to fund its OH initiatives.

## 4 Discussion

The R-OHCM has progressed in steering political commitment from Ministerial level during two consultation meetings in Dakar and Abuja. However, there is still inadequate awareness among delegates of Member States, about the operationalization of OH within the ECOWAS region which is likely to hamper on their long-term commitment. This therefore emphasizes the need for recurrent advocacy to the different national stakeholders to secure political buy-in for longer term [12]. While some of the strategic documents already exist, it would be important to have a harmonized protocol for sharing information between Member States and sectors in the future as it has proven to be effective elsewhere in maintaining rapid detection and response to previous outbreaks [23]. There is also a need to develop operational guidelines and governance documents for the R-OHCM.

West Africa has demonstrated profound effort in adopting the OH approach toward achieving global health security [16] but is presently deterred by challenges such as limited skilled One Health workforce, especially in the animal and environmental health sectors [17]. This can be attributed to failure to incorporate OH contents in all pre-service and in-service trainings in key sectors at country-levels and the inadequate collaboration between animal and environmental health experts with the NPHI on OH activities [18]. One Health Central and East Africa (OHCEA) has made effort to harmonize OH competency modules and curriculum to build capacity in OH across the Southern, Eastern and parts of Western Africa using a uniform curriculum [[19], [20]]There is still a need for the R-OHCM to focus more on plans for advocacy and capacity building for adequate and skilled OH workforce in the region while tailoring effort toward adequate funding and incorporating the developed workforce into OH activities in West Africa. Furthermore, access to quality data is problematic and arises from the inadequate and fragmented surveillance systems across sectors. There is also verticality of the different sectors and each sector still functions separately with no harmonization thus resulting in fragmented data sets that limit information sharing and crosscutting analysis. Amidst these challenges, it was recommended to ECOWAS to leverage on the existing DHIS-2 reporting systems to improve regional surveillance and harmonize reporting tools.

OHCM need unified and large-scale funding which is not only hard to acquire but also difficult to coordinate leading to the preference for programme-specific initiatives [21]. The different departments within the ECOWAS Commission have individual departmental funding, but there is no funding dedicated for the Secretariat role from the Commission to an OH agenda. Having a direct OH budget as domestic funding is essential for sustainability. The availability of funds from donor projects has greatly supported OH activities and maintained the effort toward implementation of OH in West Africa [18]. Funding from external donors often have a single-disease focus, not on strengthening the systems but are tailored toward targeted objectives especially in emergencies, which the R-OHCM often takes advantage to support OH Plans and other OH-related initiatives [[17], [19], [22]].

### 4.1 Future directions

To enhance the operationalization of the R-OHCM, there is a need to urgently develop a strategic plan and other governance documents (legal framework and M&E manual) to commit ECOWAS in implementing the OH approach.

Some other key interventions suggested for enhancing the ROHCM are to:

• Support leadership and strategic advocacy for stronger engagement of all sectors in implementing One health agenda through greater networking and collaborations with policy makers and other key stakeholders for mobilizing resources – national level (Public and private sector) and from donors;
• Strengthening the development of the Regional Center for Surveillance and disease Control and the network National Public Health Institutes based on the One Health framework as the One Health secretariat both at national and regional level
• Strengthening collaboration with other regions in Africa and the world to advance global initiatives in One Health
• Strengthen ECOWAS institutional capacities for OH implementation (both individual and organizational capacities) especially in the operationalization of One Health taskforces
• Build robust and well-prepared health systems toward Universal Health Coverage including a strong workforce and research capacities in OH
• Identify and strengthen “Center of excellence” as pockets of effectiveness in all the key sectors (Human, Animal and Environment) and One Health champions OH champions and centers of excellence
• Develop innovative strategies to increase awareness of policymakers on the successes and remaining challenges of OH implementation.
• Better integration of gender and equity considerations in promoting One Health in West Africa.

### 4.2 Limitations of the study

Challenges encountered during this review included limited access to some institutional papers and few peer reviewed publications on One Health at the ECOWAS regional level. However, the findings of this study are still valid and will inform further research, policies and practices on the implementation of R-OHCM in West Africa and also orient other regions aiming to establish a R-OHCM.

## 5 Conclusion

Having a functional and operational OHCM in the ECOWAS region depends strongly on the commitments of the ECOWAS Commission, all relevant sectors from countries and partners in the operationalization of a shared vision for advancing global health security. This scoping study identified the need for institutional changes and ownership of the R-OHCM. However, these will not drive OH forward in the absence of adequate funding and resource allocation. While major relevant components of the R-OHCM are already in place, the need to strengthen OH collaboration across sectors at the regional level to support the initiative at national levels remains.

## Conflict of interests

All Authors declared not competing interest.

## Authors' contributions

Conceptualization: VKL

Data curation: VKL, NCA

Formal analysis: VKL, NCA, SN, CDU

Funding acquisition: VKL

Investigation

Methodology: VKL, NCA, CDU, IS, SN

Project administration: VKL, SO

Resources

Software

Supervision: VKL

Validation: VKL, IS, VI

Visualization

Writing – original draft: VKL, NCA, CDU

Writing – review and editing: VKL, NCA, SN, BYK, VI, NO, CDU, CBB, IS, SO, EO
